# Supplementary material for: Vitamin D Modified DSS-Induced Colitis in Mice via STING Signaling Pathway
Source: Biology (Basel). 2025 Jun 18;14(6):715. doi: 10.3390/biology14060715 (PMC12190092; doi:10.3390/biology14060715)
Supplement: Supplementary file 1 [file biology-14-00715-s001.zip › Supplementary Materials S1: 16s-report of gut microbiota/Groups/diff_analysis/TaxaMarkers/Genus.Groups.sig.boxplot.pdf]

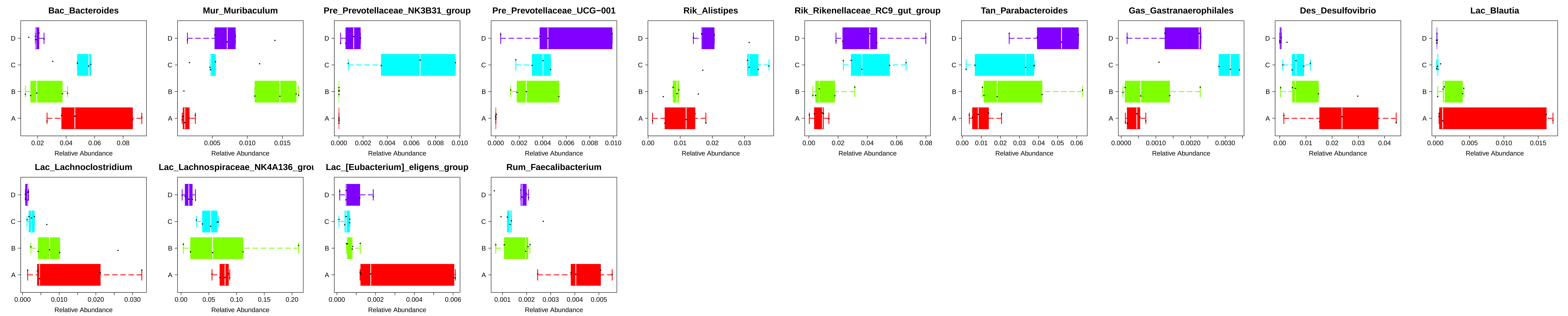
Bac_Bacteroides
Mur_Muribaculum
Pre_Prevotellaceae_NK3B31_group
Pre_Prevotellaceae_UCG−001
Rik_Alistipes
Rik_Rikenellaceae_RC9_gut_group
Tan_Parabacteroides
Gas_Gastranaerophilales
Des_Desulfovibrio
Lac_Blautia
Lac_Lachnoclostridium
Lac_Lachnospiraceae_NK4A136_grou
Lac_[Eubacterium]_eligens_group
Rum_Faecalibacterium
D
C
B
A
Relative Abundance
0.02 0.04 0.06 0.08
0.005 0.010 0.015
0.000 0.002 0.004 0.006 0.008 0.010
0.00 0.01 0.02 0.03
0.00 0.02 0.04 0.06 0.08
0.00 0.01 0.02 0.03 0.04 0.05 0.06
0.0000 0.0010 0.0020 0.0030
0.00 0.01 0.02 0.03 0.04
0.000 0.005 0.010 0.015
0.000 0.010 0.020 0.030
0.00 0.05 0.10 0.15 0.20
0.000 0.002 0.004 0.006
0.001 0.002 0.003 0.004 0.005
